# A Stability Indicating HPLC Assay Method for Analysis of Rivastigmine Hydrogen Tartrate in Dual-Ligand Nanoparticle Formulation Matrices and Cell Transport Medium

**DOI:** 10.1155/2018/1841937

**Published:** 2018-03-01

**Authors:** Naz Hasan Huda, Bhawna Gauri, Heather A. E. Benson, Yan Chen

**Affiliations:** School of Pharmacy and Biomedical Sciences, Curtin Health Innovation Research Institute, Curtin University, Perth, WA 6845, Australia

## Abstract

The objective of this study was to develop and validate a method for quantitative analysis of rivastigmine hydrogen tartrate (RHT) in dual-ligand polymeric nanoparticle formulation matrices, drug release medium, and cellular transport medium. An isocratic HPLC analysis method using a reverse phase C_18_ column and a simple mobile phase without buffer was developed, optimised, and fully validated. Analyses were carried out at a flow rate of 1.5 mL/min at 50°C and monitored at 214 nm. This HPLC method exhibited good linearity, accuracy, and selectivity. The recovery (accuracy) of RHT from all matrices was greater than 99.2%. The RHT peak detected in the samples of a forced degradation study, drug loading study, release study, and cellular transport study was pure and free of matrix interference. The limit of detection (LOD) and limit of quantification (LOQ) of the assay were 60 ng/mL and 201 ng/mL, respectively. The method was rugged with good intra- and interday precision. This stability indicating HPLC method was selective, accurate, and precise for analysing RHT loading and its stability in nanoparticle formulation, RHT release, and cell transport medium.

## 1. Introduction

There is a worldwide increase in the prevalence of brain diseases such as Alzheimer's disease (AD), Parkinson's disease, and stroke due to the increase of the aging population. Consequently, there is an increase in demand for effective treatments for these diseases. Rivastigmine hydrogen tartrate (RHT) was approved by the US FDA in 2000 for the treatment of mild-to-moderate dementia of either Alzheimer's type or related to Parkinson's disease [1]. RHT is a cholinesterase inhibitor that inhibits both acetylcholinesterase (AChE) and butyrylcholinesterase (BuChE) enzymes responsible for the degradation of acetylcholine (ACh) into nonfunctional metabolites. Among the many chemical changes that the brain encounters during AD, depletion of ACh is one of the earliest and biggest changes. RHT increases the central cholinergic function by enhancing the ACh level in mild-to-moderate AD patients and inhibits deposition of amyloid plaques in the brain, slowing down the mental decline [2–6].

RHT has been reported to improve or maintain patients' cognitive function, global function, behaviour, and day-to-day activities [7, 8]. It is commercially available as capsules, oral solution, and patches. However, the current therapeutic regimen of RHT demands frequent dosing, and cholinergic side effects are common. Like other CNS drugs, treatment efficacy of RHT is primarily restricted, not by the drug's inherent potency but by its ability to cross the blood-brain barrier (BBB) into the brain due to its hydrophilic nature [9].

To overcome the challenge of RHT transport into the brain, it has been formulated into nanoparticles (NPs) since 2008 [9]. NPs act as a drug carrier to provide targeted delivery of a concentrated payload to, and sustained release at, the target site for therapeutic action. The targeting ability of NPs is influenced by surface ligands that interact specifically at receptors on the target site. This targeting approach is receiving increased attention for the development of highly efficacious therapeutics with minimal side effects that can be used in a wide range of diseases where ideal pharmaceutical options are currently limited. Accurate and efficient analytical methods that can facilitate the formulation development and evaluation process are essential. Our group recently developed dual-targeting ligand NPs for the brain delivery of RHT [10, 11]. This was facilitated by the development of an analytical method for quantitation of RHT loading in a complex formulation and relevant cellular and stability studies.

In the past, the quantitative determination of RHT has been reported using several analytical techniques such as spectrophotometry [12, 13], HPLC [14–17], gas chromatography-mass spectrometry (GC-MS) [18–20], and liquid chromatography-mass spectrometry (LC-MS) [21–25]. For example, Fazil et al. [12] determined drug loading and encapsulation efficiency by measuring the amount of free RHT in the NPs supernatant using a UV spectrophotometer. The group used the same technique to quantify RHT in phosphate-buffered saline (PBS) in their in vitro permeability studies. Nagpal et al. [13] employed spectrophotometry to measure drug loading, entrapment efficiency, and RHT release from NPs in PBS. However, spectrophotometry is not selective and cannot separate formulation excipients, impurities, or degradation products from the drug itself.

HPLC methods developed for RHT commonly employ a buffer in the mobile phase and special columns, such as Kromasil C_8_, XTerra RP18, and 5C_18_-MS, with either UV or fluorescence detection for the analysis of RHT in different samples [14–17, 26–29].  Table 1 summarises the HPLC methods reported to date for separation and quantification of RHT in different sample matrices.

Although HPLC coupled with mass spectrophotometry (MS) is suitable or desirable for separation and quantification of RHT in biological samples (rat, canine, and human plasma, and rat brain and urine) [30], for initial formulation development and evaluation, a fast, economic, and simple yet selective and accurate HPLC method is preferred. Furthermore, it is advantageous if the developed method is also directly applicable for HPLC-MS analysis. Therefore, the objective of the current study was to develop and validate a simple, fast, sensitive, selective, and accurate HPLC method for the quantitative analysis of RHT loading in a dual-ligand NP formulation, its release and cellular transport, and its stability profile under different stressed conditions. Development criteria were that the chromatography should be achieved on a commonly used C_18_ column and that the mobile phase was without a buffer, thus allowing direct translation to HPLC-MS.

## 2. Experimental

RHT (purity ≥99.2%) was purchased from Innochem Technology Co., Ltd. (Beijing, China). HPLC grade acetonitrile (ACN) (purity ≥99.9%) was obtained from Thermo Fisher Scientific (Scoresby, Australia). Hanks' balanced salt solution (HBSS), 4-2-hydroxyethylpiperazine-1-ethanesulfonic acid (HEPES) (purity ≥ 99.5%), trifluoroacetic acid (TFA) (purity ≥ 99.0%), and phosphate-buffered saline (PBS) pouches were purchased from Sigma-Aldrich (Castle Hill, Australia). D-glucose anhydrous, HCl (32% w/v), and hydrogen peroxide (H_2_O_2_, 30% w/v) were obtained from Ajax Finechem Pty Ltd. (Taren Point, Australia). NaOH (purity ≥ 98.0) was obtained from BDH Laboratory Supplies (Poole, England). The transport buffer (HBSS-P) was prepared from HBSS containing 10 mM HEPES and 20 mM glucose. Ultrapure (type 1) water was generated using a Milli-Q System (Merck Millipore, Bayswater, Australia).

### 2.1. Nanoparticle Preparation and Characterisation

The dual-ligand PLGA-based NPs were prepared by a double emulsion solvent evaporation technique [31–33]. RHT was loaded in the NPs as a model drug. The NPs formulation was optimised to achieve optimum particle size for brain drug delivery and maximal drug loading in the NPs. The optimised NPs formulation was evaluated for in vitro characteristics including particle morphology, size, zeta potential, drug loading efficiency, release profile, and stability studies. The developed HPLC method was used for quantitative analysis of RHT loading in the NPs formulation, release and cellular transport, and stability profile under different stressed conditions.

### 2.2. Chromatographic Conditions

The HPLC system was an Agilent® 1200 instrument (Agilent Technologies, Mulgrave, Australia) with a degasser (G1379B), a binary pump (G1312A), and an autosampler (G1329A) with thermocontrol unit (G1330B) and VWD (G1314B), Waters® 1122/WTC-120 external column heater (Waters Australia Pty Ltd, Rydalmere, Australia), and a diode-array detector (DAD, G1315B). Data acquisition and processing were carried out with Agilent ChemStation® software version B.04.03 SP1.

Samples were maintained at 4°C in the autosampler prior to analysis. An Apollo C_18_ column, 5 *µ*m particle size, 150 mm × 4.6 mm (Grace Davison Discovery Sciences, Baulkham Hills, Australia), was maintained at 50°C. All analyses were conducted with an isocratic mode with a 1.5 mL/min flow rate of the mobile phase (20% v/v ACN in water containing 0.1% TFA, prefiltered through a 0.2 *µ*m hydrophilic nylon filter: Merck Millipore, Bayswater, Australia) and injection volume of 50 *µ*L. The detection of RHT was monitored at an UV wavelength of 214 nm.

### 2.3. Forced Degradation Studies

Forced degradation studies were conducted according to published protocols to confirm the selectivity of the developed assay method [34–36]. RHT (25 *µ*g/mL) was used for all degradation studies. For acid decomposition (hydrolysis) studies, RHT solution was prepared in 2 N HCl and incubated for 48 h at 37° and 60°C. For base hydrolysis studies, RHT solution was prepared in 0.5 N NaOH and incubated at 37°C and 60°C up to 48 h. Both the acid and alkaline samples were cooled to RT and neutralised before analysis by HPLC.

The stability of RHT in water was assessed with RHT solution (25 *µ*g/mL) incubated at 37° and 60°C for 48 h, whereas the effect of RHT oxidation was determined by incubating RHT for 48 h at 37° and 60°C in 30% H_2_O_2_.

All degraded samples were analysed by HPLC, and RHT peak purity was evaluated using a diode-array detector by obtaining five UV spectra across the peak. The similarity among these five spectra was determined and reported using ChemStation software to determine the peak purity. Coelution of any degraded product with the drug peak would make the peak impure, resulting in dissimilar UV spectra. The software also reported whether the peak purity in each spectrum was within the automatically set threshold limit.

### 2.4. Method Validation

The developed HPLC method was validated with respect to selectivity, linearity, precision, accuracy, limit of detection (LOD), and limit of quantification (LOQ) in accordance with the International Council for Harmonisation (ICH) Guidelines for Validation of Analytical Procedures, Q2B [37] and the United States Pharmacopeia and the National Formulary (USP 37-NF 32) [38].

#### 2.4.1. Selectivity

Forced degradation study samples were used to assess the selectivity of the method. Supernatants of both blank NPs (dual-ligand NPs without any loaded drug) and RHT-loaded dual-ligand NPs were diluted 200 times in the mobile phase and injected into the HPLC to study whether any interfering peaks coeluted at or near the drug peak. Similarly, the matrix interference was also investigated using (i) PBS medium collected from release study control (dual-ligand NPs without any loaded drug) and (ii) uptake/transport study buffer (HBSS-P) comprising HBSS containing 10 mM HEPES and 20 mM glucose (collectively the “experimental media”).

#### 2.4.2. Linearity

The linearity of the developed assay method was assessed in two different media: 0.3% vitamin E-TPGS (solvent for dispersion of dual-ligand nanoparticle) and PBS (release study medium). A range of concentrations of RHT solutions (0.1 to 2 mg/mL) was prepared in 0.3% vitamin E-TPGS from a stock solution (2 mg/mL RHT in 0.3% vitamin E-TPGS). Each solution was then diluted 200 times (50 *µ*L into 10 mL) with the mobile phase to obtain the final RHT standard concentrations of 0.5, 1, 2, 3, 4, 5, 6, 7, 8, 9, and 10 *µ*g/mL. These standards were injected into the HPLC column, in duplicate. Another set of standards with the same concentration range was prepared by diluting an RHT stock solution (1 mg/mL) in type 1 water with 10 mM PBS (pH 7.4). Again, these standards were injected into the HPLC column in duplicate. Average peak area data were plotted against corresponding standard concentrations using Microsoft® Excel 2016 to construct the standard calibration curve. The linearity was established by calculating the *R*^2^ value.

#### 2.4.3. Precision

The precision of the proposed method was determined by injecting four RHT concentrations (1, 4, 6, and 10 *µ*g/mL) in the experimental media, six times into the HPLC. The relative standard deviation (RSD) values were calculated for all concentrations.

#### 2.4.4. Limit of Detection (LOD)

The LOD was determined as the drug concentration that produced a signal three times greater than the baseline noise level. Two blank solvents, namely, (1) 0.3% vitamin E-TPGS diluted 200 times in the mobile phase and (2) 10 mM PBS, pH 7.4, were injected six times to determine the average noise levels. Standard RHT solutions prepared in the mobile phase were analysed and calibration curves constructed by plotting average peak heights against the corresponding concentrations. The LOD was calculated by the following formula:

(1) $$\begin{array}{c} \text{LOD}=3\times \frac{\text{Peak height of noise}}{\text{Slope of calibration curve constructed by peak height versus conc}}. \end{array}$$

#### 2.4.5. Limit of Quantification (LOQ)

Using the same data, the LOQ was determined as the concentration with the signal at ten times greater than the baseline noise level. The LOQ was calculated by the following formula:

(2) $$\begin{array}{c} \text{LOQ}=10\times \frac{\text{Peak height of noise}}{\text{Slope of curve constructed by peak height versus conc}}. \end{array}$$

#### 2.4.6. Intra- and Interday Repeatability (Ruggedness)

Three standard concentrations of RHT (low, medium, and high) in each solvent (0.3% vitamin E-TPGS diluted 200 times in the mobile phase and 10 mM PBS, pH 7.4) within the calibration curve were selected. The intra- and interday repeatability of the method was assessed by analysing these 1, 5, and 10 *µ*g/mL RHT standards, in triplicate, at different time points in the same day and on two different days. In addition, the ruggedness study was conducted by analysing another set of RHT standards of the same concentrations by a second analyst on a different day. The RSD was calculated for each analysed concentration and compared with the nominal limit to evaluate the intra- and interday repeatability of the method and ruggedness.

#### 2.4.7. Accuracy

The accuracy of the developed method was determined in two media to assess the interference of the dual-ligand NPs formulation matrices and solvents. Firstly, a batch of drug-free dual-ligand NPs (blank NPs) was prepared, and the supernatant was collected during the last step of the preparation. The supernatant was then spiked with RHT to obtain a solution of RHT (2 mg/mL). This stock solution was diluted with the same supernatant medium to prepare five RHT solutions with concentrations of 0.1, 0.4, 0.8, 1.2, and 1.6 mg/mL. These solutions were diluted 200 times with the mobile phase to obtain the final drug concentrations of 0.5, 2, 4, 6, 8, and 10 *µ*g/mL and injected into the HPLC column in triplicate. This procedure mimics the method of sample preparation for the determination of RHT loading in dual-ligand NPs.

Secondly, an in vitro release study of the blank dual-ligand NPs was carried out at pH 7.4 in PBS (mimicking the NP matrix in the release medium). 3 mL of blank dual-ligand NPs suspension was loaded in a dialysis tube (MWCO 12000), sealed, and placed in a 60 g amber glass jar containing 50 mL prewarmed PBS at 37°C. The setup was placed on an orbital shaker at 37°C and horizontally shaken at 100 rpm. Release medium from outside the dialysis bag was collected after 24 h, spiked with RHT standard solution (200 *µ*g/mL) prepared in type 1 water to get the final RHT concentrations of 2, 4, 6, 8, and 10 *µ*g/mL, and injected into the HPLC column in triplicate. The concentration of RHT in all samples was determined against RHT standards prepared in the mobile phase.

The method accuracy was determined by calculating the percentage of recovery (measured concentration over the added concentration) in each case.

## 3. Results and Discussion

The NP formulation was optimised to achieve the particle size between 70 nm and 200 nm as well as to achieve the highest possible drug loading in the NPs. The optimised RHT-loaded dual-ligand NPs were found to have negative zeta potential (−24.3 ± 2.5 mV) and narrow size distribution (139.5 ± 3.9 nm) ideal for targeting the BBB.

### 3.1. Development and Optimisation of HPLC Method

**Figure 1:**
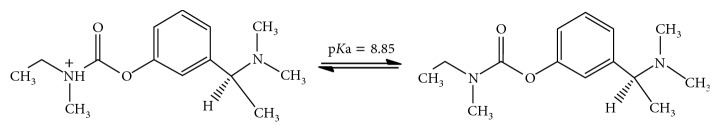
Ionisation of rivastigmine tartrate.

The HPLC method for separation and quantification of RHT in PBS (pH 7.4) and NP supernatant (containing 0.3% vitamin E-TPGS) was developed and validated. An appropriate combination of the column type, column temperature, mobile phase composition and flow rate, injection volume, and detection system was studied to produce a simple, fast, economic, and yet selective and accurate assay method. We validated 50 *µ*L injection volume as the maximum injection volume for future application in analysis of biological samples. The UV wavelength of 214 nm was selected for the detection of the compound based on the UV spectrum of RHT. A lower wavelength (209 nm) produced a much stronger drug signal, but higher background noise made this approach impractical. The mobile phase composition was developed based on the solubility and p*K*a of RHT (8.85) [39]. An acidic condition (pH 2.6) was considered necessary to keep all RHT molecules ionised (Figure 1). At this lower pH, the silane groups of the C_18_ column were also fully protonated, leading to weak interaction with RHT, thereby shortening the elution time. TFA was used to provide a good peak shape and avoid the use of buffer salts that may precipitate due to an interaction with formulation excipients. In addition, eliminating a buffer allows the method to be easily adapted for LC-MS analysis of RHT in the future. The initial trial mobile phase composed of ACN and water (50 : 50 v/v) containing 0.1% TFA at a flow rate of 1 mL/min resulted in the RHT eluting with the solvent front. Consequently, the organic phase was optimised at a ratio of 20 : 80 (v/v) for ACN : water to produce the best peak shape and separation. The flow rate was increased to 1.5 mL/min, and column temperature was maintained at 50°C to facilitate separation, sharpen the peaks, and reduce the retention time to 6.8 min. Mullangi et al. [16] also employed a similar mobile phase composed of ACN and water (26 : 74 v/v) and acidic pH of 4.5 for the analysis of RHT. However, our developed method utilises a less-expensive shorter column and a shorter run time, thereby providing economic benefits.

### 3.2. Forced Degradation of RHT and Selectivity

The main aim of the forced degradation studies of RHT was to assess the selectivity of the analytical method. According to the MSDS supplied by the manufacturer, RHT is chemically stable under normal conditions but incompatible with strong acids, bases, and oxidising agents. No light sensitivity data were provided in the MSDS; however, RHT was reported to be stable when exposed to light for at least ten days [27]. In our investigation, various stress conditions were employed to simulate any possible degradation that might occur during the NPs preparation and in vitro characterisation experiments. RHT was subjected to hydrolysis (acidic, alkaline, and neutral pH) and oxidation. Our results (Table 2) showed a similar degradation pattern for RHT as per the published literature [27]. We found that RHT was most prone to base degradation, showing maximum degradation after 48 h incubation at 37°C, compared to acidic, oxidative, and hydrolysis in water conditions. The drug demonstrated excellent stability against hydrolysis conditions at neutral pH under both test temperatures, but oxidised easily at 37°C, with complete degradation after 48 h incubation at 60°C. RHT stability in 2 N HCl was relatively good with 2.2% degradation at 37°C over 48 h.

**Figure 2:**
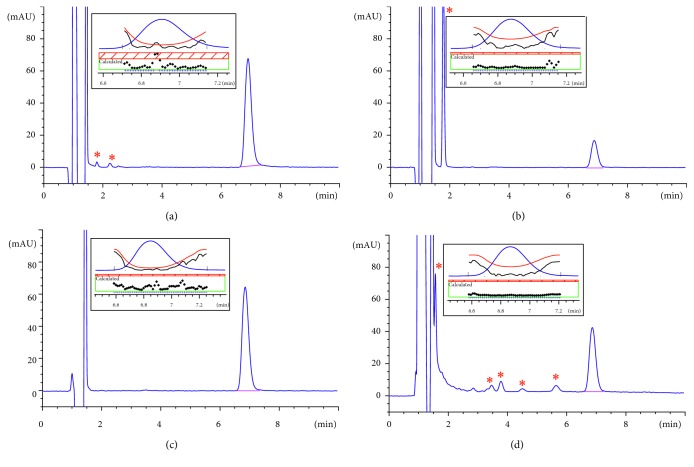
HPLC chromatogram of RHT under various stress conditions conducted at 37°C for 48 hours. (a) Acid degradation, (b) alkali degradation, (c) hydrolysis, and (d) oxidation. Analysis of RHT was not interfered by the degradation products (∗). Peak purity reports are shown in the insets, confirming that the RHT peaks are pure and the purity factors are within the calculated threshold limit.

Figure 2 illustrates that our developed HPLC assay method is capable of separating RHT from all degradation products and that the RHT peak obtained at 6.8 min is pure. Peak purity analysis was conducted using the default settings of the ChemStation software without any manual data entry. The peak purity of degraded products was not checked because we were only interested in assessing the method's capability of resolving the pure RHT peak. The eluted RHT peak was well separated from the degraded products (retention time < 6 min). Thus, the developed method was selective and can be used as a stability indicating method for the analysis of RHT concentration in various samples including stability samples.

**Figure 3:**
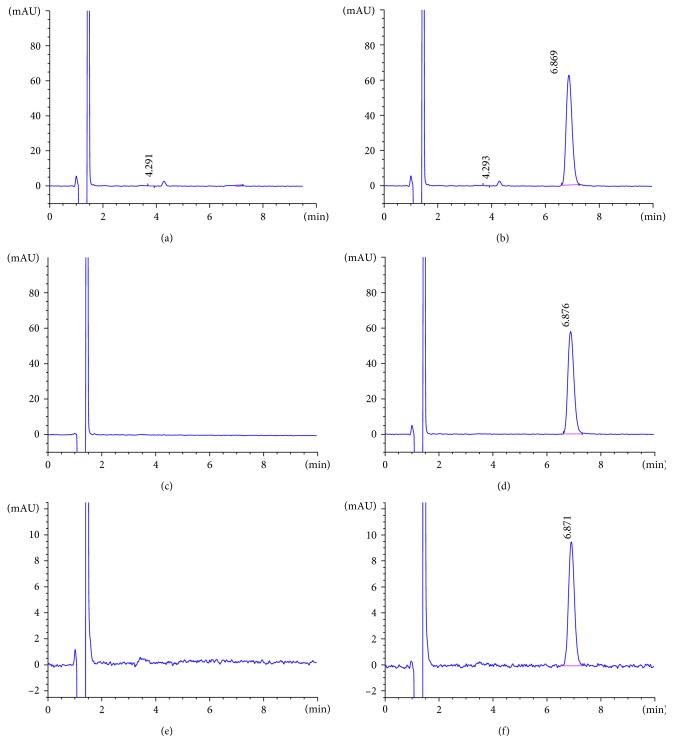
HPLC chromatograms illustrating absence of any matrix interfering peak around the RHT retention time (6.9 minutes). Chromatograms of (a) empty dual-ligand NPs matrix, (b) RHT-loaded dual-ligand NPs matrix, (c) release medium after 24-hour release study of empty dual-ligand NPs, (d) release medium after 24-hour release study of RHT-loaded dual-ligand NPs, (e) cell transport medium, and (f) released RHT from NPs in cell transport medium after the transport study.

As our RHT samples of interest are from dual-ligand nanoparticle formulation, release, and cellular transport studies, it is important that the matrices present in those samples do not interfere with the RHT quantitation. Therefore, further selectivity studies were carried out to confirm that the developed HPLC method has the capability to generate “true results” that are free from matrix or medium interference. The HPLC spectra in Figure 3 indicate that there was no peak around the RHT retention time (6.8–6.9 min) in any of the experimental media: (i) supernatant of dual-ligand NPs without any loaded drug (after 200 times dilution with the mobile phase), (ii) PBS medium following release study of empty dual-ligand NPs, and (iii) cellular transport study medium (HBSS-P). This demonstrates that there was no matrix interference and the method is selective or specific for analysis of RHT under various conditions.

### 3.3. Linearity

The detector response to various concentrations of RHT in two media produced a linear relationship. For 50 *µ*L injections of RHT in vitamin E-TPGS (following 200 times dilution with the mobile phase), the regression plot demonstrated a nearly perfect linear relationship (coefficient of variance was 0.9999) over the concentration range of 0.5–10 *µ*g/mL that covered the concentrations encountered in the RHT loading analysis. The same concentration range of RHT in PBS also demonstrated a good linear relationship with a coefficient of variance of 0.9998.

### 3.4. Precision

The precision study was also conducted with the three media used in the selectivity study (Table 3). All RSD values were well below the nominally acceptable level of ≤2% [40]. Even at the low concentrations of RHT (1*μ*g/mL), the RSD of 1.04–1.28% was achieved, demonstrating that the method is precise.

### 3.5. LOD and LOQ

The LOD of an analytical procedure is the lowest detectable amount of an analyte in a sample but not necessarily a quantifiable value. For the current method, the lowest detectable concentration of RHT in both solvent systems was 60 ng/mL.

The LOQ is the lowest amount of the drug in the sample that can be confidently quantified using the method. For the current method, the lowest quantifiable concentration of RHT in both solvent systems was 201 ng/mL. The LOD and LOQ is in a comparable range or even better than other published methods [14, 27], and this method can also meet the analytical requirements of dual-ligand NP formulation development and evaluation.

### 3.6. Intra- and Interday Repeatability

The intra- and interday repeatability data are shown in Table 4. All RSD values of repeated analysis were within the acceptable limit of ≤2% [40]. The ruggedness study performed by different analysts also demonstrated similar trend with RSD values below 2% (data not shown). These results suggest that the developed method is rugged.

### 3.7. Accuracy

The accuracy of a method demonstrates that the assay can accurately quantify the molecule(s) of interest in the presence of other possible interfering components such as excipients, reactions components, release medium, and cellular transport medium. The accuracy of the proposed method was calculated as percentage recovery from the six concentrations covering the entire RHT concentration range within the calibration curve. RHT was successfully recovered from all samples in three experimental media (Table 5) with an accuracy of 99.5 ± 1%, which is within the acceptable range [40]. This suggests that none of the matrices in the dual-ligand nanoparticle formulation or medium/buffer is interfering with the assay of RHT. It can be concluded that the developed HPLC assay method can be used to produce accurate data.

## 4. Conclusion

To assist the development of dual-ligand NP formulations for brain drug delivery, we have developed a simple, fast, accurate, and reliable HPLC method for RHT analysis during the formulation development and evaluation. This HPLC method has been validated for analysis of RHT loading in dual-ligand NPs preparations, in vitro drug release, and cellular transport studies. The chromatographic separation was achieved using a C_18_ column maintained at 50°C and an isocratic mobile phase consisting of TFA containing ACN and water with a flow rate of 1.5 mL/min. The method exhibited good linearity over the assayed concentration range and good intra- and interday precision. The developed HPLC method is accurate, selective, and rugged for RHT analysis with good detection and quantification limits and is suitable for its intended use. This stability indicating analytical method can be adapted easily to analyse RHT in pharmaceutical formulations and biological matrices and for the future use in HPLC-MS analysis.

## Figures and Tables

**Table 1:** Summary of published HPLC conditions for RHT determinations.

Column	Sample matrix	Mobile phase	Flow rate	Detection technique	Analysis time	Detection limits	Reference
Waters Spherisorb silica	Human plasma	Acetonitrile-50 mM aqueous sodium dihydrogen phosphate (17 : 83 v/v, pH 3.1)	1.3 mL/min	UV: 200 nm	6 minutes	LOD: 0.2 ng/mL	[26]
						LOQ: 0.5 ng/mL	
Inertsil ODS-3V C_18_	Rat plasma and brain	Ammonium acetate buffer (20 mM, pH 4.5) and acetonitrile 74 : 26 (v/v)	1.0 mL/min	Fluorescence detector, Ex/Em wavelength: 220/293 nm	16 minutes	LOD: not given	[16]
						LOQ: 10 ng/mL	
XTerra RP18 C_18_	Raw material	10 mM sodium-1-heptane sulphonate (pH 3.0) and acetonitrile 72 : 28 (v/v)	1.0 mL/min	UV: 217 nm	13 minutes	LOD: 100 ng/mL	[27]
						LOQ: 300 ng/mL	
Monomeric C_18_	Rat plasma	Acetonitrile and 20 mmol/L phosphate buffer, pH 3.0 (25 : 75)	1.0 mL/min	Fluorescence detector, Ex/Em wavelength:220/293 nm	20 minutes	LOD: not given	[28]
						LOQ: 25 ng/mL	
5C_18_-MS	Capsule	Methanol and water (90 : 10)	1.0 mL/min	UV: 217 nm	Not given	LOD: not given	[29]
						LOQ: 10.9 *µ*g/mL	
Kromasil C_8_	Liposomes	20 mmol·L^−1^ phosphate buffer (pH 3.0) and acetonitrile (75 : 25%, v/v)	1.0 mL/min	UV: 210 nm	20 minutes	LOD: not given	[17]
						LOQ: 10 ng/mL	
C_18_	Solid lipid nanoparticles	Acetonitrile and potassium dihydrogen orthophosphate buffer (pH 6.0) (20 : 80 v/v)	1.0 mL/min	UV: 215 nm	Not given	LOD: not given	[14]
						LOQ: 1 *µ*g/mL	
ODS C_18_	Liposomes	Acetonitrile : water (20 mM NaH_2_PO_4_·2H_2_O, 10 mM Na_2_HPO_4_·12H_2_O) (25 : 75, v/v)	1.0 mL/min	UV: 218 nm	Not given	LOD: not given	[15]
						LOQ: not given	

**Table 2:** Summary of findings in RHT-forced degradation studies.

Forced degradation condition	Temp. (°C)	Incubation duration (hrs)	Remaining percentage
Acid hydrolysis: RHT in 2 N HCl	60	48	87.4
	37	48	97.8
Base hydrolysis: RHT in 0.5 N NaOH	60	2	80.9
	60	48	0.0
	37	48	29.7
Hydrolysis: RHT in water	60	48	99.2
	37	48	99.7
Oxidation: RHT in 30% (w/v) H_2_O_2_	60	48	0.0
	37	48	79.6

**Table 3:** The precision of the HPLC method for determination of RHT.

RHT conc. (*µ*g/mL)	RHT in NPs matrix		RHT in release medium		RHT in cell transport medium	
	Average RHT peak area (mAU × sec)	RSD (%)	Average RHT peak area (mAU × sec)	RSD (%)	Average RHT peak area (mAU × sec)	RSD (%)
1	36.50	1.04	36.22	1.28	36.02	1.06
4	145.97	0.59	145.82	0.67	144.24	0.66
6	219.73	0.49	220.98	0.28	218.64	0.48
10	370.20	0.17	369.30	0.19	369.15	0.23

**Table 4:** Intra- and interday repeatability of RHT analysis in NPs matrix, release medium, and cell transport medium.

RHT concentration^a^ (*µ*g/mL)	RHT in NPs matrix (200x diluted in mobile phase)		RHT in release medium		RHT in cell transport medium	
	Intraday RSD^b^	Interday RSD^c^	Intraday RSD^b^	Interday RSD^c^	Intraday RSD^b^	Interday RSD^c^
1	0.69	1.32	0.73	0.64	0.94	1.24
5	0.92	0.92	0.65	1.11	0.51	1.11
10	0.49	1.19	0.35	1.02	0.15	0.95

^a^Each concentration was analysed in triplicate (*n*=3); ^b^the analyses were carried out at 0, 3, and 8 hrs on the same day, and all data were included in the calculation; ^c^the analyses were carried out at days 1 and 2, and all data were included in the calculation.

**Table 5:** Accuracy data for RHT in NPs matrix, release medium, and cell transport medium.

Prepared RHT concentration (*µ*g/mL)	RHT in NPs matrix		RHT in release medium		RHT in cell transport medium	
	Measured concentration (*µ*g/mL)	Recovery (%)	Measured concentration (*µ*g/mL)	Recovery (%)	Measured concentration (*µ*g/mL)	Recovery (%)
0.50	0.49 ± 0.13	98.0 ± 0.2	0.49 ± 0.11	98.4 ± 0.1	0.49 ± 0.16	98.6 ± 0.2
2.00	1.99 ± 0.16	99.5 ± 0.3	2.01 ± 0.10	100.5 ± 0.3	2.00 ± 0.12	99.8 ± 0.2
4.00	3.96 ± 0.19	99.0 ± 0.3	4.04 ± 0.24	101.0 ± 0.2	3.95 ± 0.31	98.7 ± 0.3
6.00	5.97 ± 0.11	99.5 ± 0.1	5.95 ± 0.25	99.2 ± 0.2	5.93 ± 0.15	98.8 ± 0.2
8.00	8.03 ± 0.16	100.4 ± 0.3	7.98 ± 0.19	99.8 ± 0.6	7.97 ± 0.16	99.6 ± 0.2
10.00	9.99 ± 0.37	99.9 ± 0.2	9.99 ± 0.15	99.9 ± 0.2	9.98 ± 0.24	99.8 ± 0.1
	Mean ± SD = 99.4 ± 0.8		Mean ± SD = 99.8 ± 0.9		Mean ± SD = 99.2 ± 0.9	
